# CRISPR Detection and Research on Screening Mutant Gene *TTN* of Moyamoya Disease Family Based on Whole Exome Sequencing

**DOI:** 10.3389/fmolb.2022.846579

**Published:** 2022-03-09

**Authors:** Yilei Xiao, Weidong Liu, Jiheng Hao, Qunlong Jiang, Xingbang Wang, Donghu Yu, Liyong Zhang, Zhaogang Dong, Jiyue Wang

**Affiliations:** ^1^ Department of Neurosurgery, Liaocheng People’s Hospital, Liaocheng, China; ^2^ Department of Geriatric Medicine, Qilu Hospital of Shandong University, Ji’nan, China; ^3^ Department of Neurosurgery, Zhongnan Hospital of Wuhan University, Wuhan, China; ^4^ Department of Clinical Laboratory, Qilu Hospital of Shandong University, Ji’nan, China

**Keywords:** moyamoya disease, *TTN*, CRISPR-Cas12a, RNF_213_, MMP3

## Abstract

Moyamoya disease (MMD) has a high incidence in Asian populations and demonstrates some degree of familial clustering. Whole-exome sequencing (WES) is useful in establishing key related genes in familial genetic diseases but is time-consuming and costly. Therefore, exploring a new method will be more effective for the diagnosis of MMD. We identified familial cohorts showing MMD susceptibility and performed WES on 5 affected individuals to identify susceptibility loci, which identified point mutation sites in the titin (*TTN*) gene (rs771533925, rs559712998 and rs72677250). Moreover, *TTN* mutations were not found in a cohort of 50 sporadic MMD cases. We also analyzed mutation frequencies and used bioinformatic predictions to reveal mutation harmfulness, functions and probabilities of disease correlation, the results showed that rs771533925 and rs72677250 were likely harmful mutations with GO analyses indicating the involvement of *TTN* in a variety of biological processes related to MMD etiology. CRISPR-Cas12a assays designed to detect *TTN* mutations provided results consistent with WES analysis, which was further confirmed by Sanger sequencing. This study recognized *TTN* as a new familial gene marker for moyamoya disease and moreover, demonstrated that CRISPR-Cas12a has the advantages of rapid detection, low cost and simple operation, and has broad prospects in the practical application of rapid detection of MMD mutation sites.

## Introduction

Moyamoya disease (MMD) is a chronic progressive, cerebrovascular, and occlusive disease of unknown etiology first reported by Suzuki in 1969 (Kuroda and Houkin, 2008). Compared with western country, the incidence of MMD is higher in China, Korea and Japan, among which MMD is the main cause of stroke in children and adolescents (Kim, 2016; Zhao et al., 2018; Deng et al., 2021). Previous studies have shown a higher incidence of moyamoya disease in East Asia, among which, particularly in China, the incidence of moyamoya disease in the north is significantly higher than in the south (Hu et al., 2017). In recent years, a number of studies have confirmed a genetic susceptibility for MMD, proposing that genetic factors play a major role in the pathogenesis of MMD (Liu et al., 2011; Morito et al., 2014; Kobayashi et al., 2015; Kim, 2016). For example, 10–15% of MMD patients have a family history, and the prevalence of these people with a family history is 30–40% higher than that of ordinary people (Kim, 2016). Therefore, it is easier to obtain potential genetic related genes through the research on family patient.

The first pathogenic gene to be associated with MMD was the ring finger protein 213 (RNF213) (Kamada et al., 2011). Moreover, two mutations within the *RNF213* gene (rs112735431 and rs148731719) were known to be associated with MMD pathogenesis in Chinese patients (Wu et al., 2012; Zhang et al., 2017; Wang Y. et al., 2020). In 2010, researchers discovered that the-1171 locus of the *MMP3* gene in Chinese Han patients was closely related to the onset of MMD (Li et al., 2010); this work also represented the first research on susceptibility genes in China. Other studies have also shown that 6–10% of Chinese MMD cases are likely to be familial in origin (Hishikawa et al., 2013). In addition, a novel missense mutation 377T > C and two polymorphisms (420A > G and 487C > T) in the TGIF gene were identified in a Taiwanese family segregated with holoprosencephaly (HPE) and moyamoya disease, speculated the possible association between TGIF mutation and MMD (Chen et al., 2006). An extensive genetic study on specific gene in MMD patients might shed light on the pathogenesis of MMD. Our previous studies have shown that specific gene mutations does not lead to inheritance of the disease. To some extent, our data can serve as a useful complement to family-based research.

With the development of high-throughput sequencing technology, WES has been increasingly utilized in the study of Mendelian diseases and complex diseases. The human exome region accounts for only 1% of the entire genomic sequence, but approximately 85% of known pathogenic mutations are located in coding regions (Manolio et al., 2009). Notably, traditional mutation site screening mostly uses Sanger sequencing or WES, which is time-consuming and costly, not being beneficial to the large-scale screening of samples. The CRISPR-Cas system is an important immune defense system of Archaea and bacteria against viral and plasmid infection (Ishino et al., 1987; Jansen et al., 2002; Mojica et al., 2005). Cas12a (cpf1) is a new type of programmable DNA enzyme found in the CRISPR system and contains an RuvC domain and a specific nuclease domain (Zhou et al., 2014). Some studies have found that Cas12a also has the ability to cut non-target DNA following cleavage of the target DNA (Gilbert et al., 2013; Qi et al., 2013). The CRISPR-Cas system has extremely high sensitivity and efficiency in the detection of nucleic acids, which has changed the process of molecular diagnosis of various diseases (Chertow, 2018).

In the pre-experiment, we verified the utility of the CRISPR-Cas12a and Sanger to detect specific gene (RNF213 and MMP3) mutations. In this study, we used WES to analyze familial cases of MMD from Chinese patients. The CRISPR-Cas12a system was used to screen the mutation loci of disease-related families and identify related genes, thereby uncovering the molecular basis of MMD.

## Methods

### Collection of Clinical Samples

We recruited MMD patients (≥18 years old and ≤70 years old, male: female = 1:1) without previous medical history. Diagnostic criteria were based on the Japanese Research Committee on moyamoya disease of the Ministry of Health, Welfare and Labour, Japan (RCMJ) criteria (Research Committee on the Pathology and Treatment of Spontaneous Occlusion of the Circle of Willis and Health Labour Sciences Research Grant for Research on Measures for Infractable Diseases, 2012). Their clinical diagnosis was confirmed by imaging with transcranial computed tomography (CT), magnetic resonance imaging (MRI), or digital subtraction angiography (DSA) along with various clinical judgments. Fasting samples of venous blood were collected from all patients and healthy control subjects separately during the same period. All subjects signed the consent form prior to entering the trial.

### Primer Design and Preparation of crRNA

Wild-type and mutant templates were designed with reference to the known mutation detection loci for the specific gene. Amplimers and crRNAs were then designed for the known mutation regions and oligonucleotides (crDNA) were synthesized. crDNA and cr-T7-F were mixed and boiled for 10 min, then the double-stranded transcription template being formed by natural cooling. The transcription template was then incubated for 16 h at 37°C under enzymatic-free conditions using the HiScribe T7 Quick High Yield RNA Synthesis Kit (NEB, Ipswich, United States). After the completion of the reaction, 2 μL of DNase 1 (TianGen, Beijing, China) was added to eliminate unreacted template before purifying the crRNA. Wild-type and mutant template sequences, amplimers, and crDNAs, were synthesized by Tianyi Huiyuan Biotechnology Co., Ltd. (Supplementary Table S1).

### Validation of the CRISPR-Cas12a Fluorescence Detection System

Fncas12a uses 5′-KYTV-3′ 999 as protospacer adjacent motif (PAM). It was chosen as the detection protein for providing more target sequence options compared with Ascas12a and Lbcas12a (Tu et al., 2017). In brief, 50 ng of template DNA was added into the detection reagent mixture containing 0.75 μM crRNA, 1.5 μM Fncas12a, 50pM of fluorescent probe, and 3 μL of NEBuffer 3.1 (NEB, Ipswich, United States). Reactions (50 μL) were then incubated at 37°C for 1h prior to fluorescence quantification. All reactions were carried out at 37°C.

### Clinical Sample Testing

Following plasma separation, DNA was extracted from venous blood samples. Thereafter, polymerase chain reactions (PCR) were performed using 50 ng of DNA as the template with specific primers (Supplmentary Table S1) at the following cycle conditions: 95°C for 5 min; 30 cycles of 95°C for 3 min; 56°C for 10 s, and 72°C for 20 s; followed by 72°C for 5 min. PCR products were then visualized by agarose gel electrophoresis and were sequenced using the Sanger method. In parallel, 1–5 μl of amplified product was used for CRISPR-Cas12a fluorescence detection.

### Collection and Selection of Samples for Whole-Exome Sequencing

We collected five samples from the familiy with clinical manifestations of the MMD phenotype from Liaocheng People’s Hospital Center from June 2020 to December 2020 (Table 1). All five family members were subjected to WES as depicted in the flow chart in Supplementary Figure S1. This study was approved by the ethics committee of Liaocheng People’s Hospital, Shandong Province. Informed consent for DNA analysis was obtained from patients in line with local Institutional Review Board (IRB) requirements at the time of collection.

**TABLE 1:** Sample information.

Specimen No.	Sex (male/female)	Patient or Normal (*P*: Patient; N: Normal)
B1	F	*P*
B2	M	N
B3	M	N
B4	M	N
B5	F	P

### Library Construction for Whole-Exome Sequencing

DNA extracted from peripheral blood was fragmented to an average size of 180–280 bp and subjected to DNA library creation using established Illumina paired-end protocols. The Agilent SureSelect Human All ExonV6 Kit (Agilent Technologies, Santa Clara, CA, United States) was used for exome capture according to the manufacturer’s instructions. The Illumina NovaSeq 6,000 platform (Illumina Inc., San Diego, CA, United States) was utilized for genomic DNA sequencing in Novogene Bioinformatics Technology Co., Ltd. (Beijing, China) to generate 150-bp paired-end reads with a minimum coverage of 10× for 99% of the genome (mean coverage of 100×).

### Whole-Exome Sequencing Data Analysis

After sequencing, base-call file conversions and demultiplexing were performed with bcl2fastq software (Illumina). The resulting fastq data were submitted to in-house quality control software to remove low quality reads; and these were then aligned to the reference human genome (hs37d5) using the Burrows-Wheeler Aligner (bwa) (Li and Durbin, 2009). Duplicate reads were marked using sambamba tools (Tarasov et al., 2015). Single nucleotide variants (SNVs) and indels were identified by samtools to generate Genome VCF (gVCF) (Li et al., 2009). Raw calls for the SNVs and INDELs were further filtered with the following inclusion thresholds: 1) a read depth > 4; 2) a root-mean-square mapping quality of covering reads that was > 30; and 3) a variant quality score > 20. Copy number variants (CNVs) were detected with CoNIFER software (Version 0.2.2) (Krumm et al., 2012). Annotation was performed using ANNOVAR (2017) (Wang et al., 2010). Annotations included minor allele frequencies from public control data sets as well as deleteriousness and conservation scores, thus enabling further filtering and assessment of the likely pathogenic variants.

### Selection of Candidate Mutation Loci

Filtering for rare variants was performed as follows. First, variants with a MAF < 0.01 in 1000 genomic data (1000g_all) (Auton et al., 2015), esp6500siv2_all, and gnomAD data (gnomAD_ALL and gnomAD_EAS); (Kim, 2016) only SNVs occurring in exons or splice sites (splicing junction 10 bp) were further analyzed since we were targeting amino acid changes; (Deng et al., 2021) synonymous single nucleotide variants (SNVs) which were not relevant to the amino acid changes predicted by dbscSNV were discarded; the small fragment non-frameshift (<10bp) indel in the repeat region defined by RepeatMasker was discarded; and (Zhao et al., 2018) variations were screened according to SIFT scores (Kumar et al., 2009), PolyPhen (Adzhubei et al., 2010), MutationTaster (Schwarz et al., 2010) and CADD (Kircher et al., 2014) software packages. Potentially deleterious variations were reserved if the scores from more than half of the four software packages identified the variations as harmful (Muona et al., 2015). Sites (>2bp) that did not affect alternative splicing were also removed. To better predict the harmfulness of each variation, we applied the classification system put forward by the American College of Medical Genetics and Genomics (ACMG). The variations were classified as pathogenic, likely to be pathogenic, of uncertain significance, likely to be benign, or benign (Richards et al., 2015). Depending upon various considerations (pedigree, homozygous, and compound heterozygous), variants were considered to be candidate causal variations. The relationship between the proband and the parents was estimated using the pairwise identity-by-descent (IBD) calculation in PLINK (Purcell et al., 2007). The share of IBD between the proband and parents for all trios ranged from 45 to 55%.

### Statistical Analysis

SPSS 17.0 software was used for statistical analysis. The qualitative data and the number of cases described in percentage, and the quantitative data were compared by independent sample *t*-test or analysis of variance. *p* < 0.05 indicates a significant difference.

## Results

### The Ability of CRISPR-Cas12a to Detect Mutations

Literature searches identified *RNF213* as a susceptibility gene for MMD. In addition, two SNP loci of *RNF213*, rs112735431 and rs148731719 have been confirmed closely related to MMD (Liu et al., 2011; Zhang et al., 2017; Wang Y. et al., 2020). crRNA was designed to detect these two SNP point mutation loci in *RNF213*. The cleavage efficiency of the crRNAs was then verified against wild-type and mutant-target DNA (Supplementary Figure S3). The fluorescence levels derived from the mutant were significantly higher than the wild type (*p* < 0.05), indicating that the CRISPR-Cas12a system constructed with the indicated crRNAs could successfully detect whether there was a mutation at this locus in clinical samples.

### Detection of *RNF213* Gene Locus by CRISPR-Cas12a and Sanger Sequencing

We collected 34 samples of patients who had been clinically diagnosed with MMD and 37 healthy control samples from Liaocheng People’s Hospital. DNA was extracted from these samples and the *RNF213* gene of samples was tested using the CRISPR along with Sanger sequencing (Supplementary Table S2). The coincidence rate of the CRISPR-Cas12a system and Sanger sequencing for detecting mutation samples was 100%, indicating that the CRISPR-Cas12a detection is accurate and highly sensitive.

### Analysis of the Correlation Between Gene (*RNF213*, *MMP3*) Mutations and MMD

First, the results of the Sanger test for RNF213 showed that there was a C > T mutation at locus rs112735431 and a G > A mutation at locus rs148731719 in the *RNF213* gene (Supplementary Figure S4A). T-tests showed that the *p* value for the rs112735431 locus mutation was < 0.05 when comparing between the case group and the healthy control group from the Liaocheng area. In contrast, there was no significant difference between the groups with respect to rs148731719 (*p* > 0.05) (Supplementary Table S3), indicating that the rs112735431 mutation within the *RNF213* gene was significant (*p* < 0.05) and that the rs112735431 was a significant mutation locus for MMD in the *RNF213* gene.

Then, we identified a base insertion mutation (rs3025058) in the *MMP3* gene (Supplementary Figure S4B). This mutation was identified by Sanger sequencing and detected in 67.6% of the 34 patients with MMD in Shandong province, and 5.4% of the 37 controls, indicating statistical significance (*p* < 0.05). The 1171 (6A/6A) mutation in the *MMP3* gene is associated with the risk of MMD. furthermore, the risk of the (6A/6A) genotype is higher than that of the (5A/6A) genotype (Supplementary Table S3.

### Whole-Exome Sequencing

**FIGURE 1:**
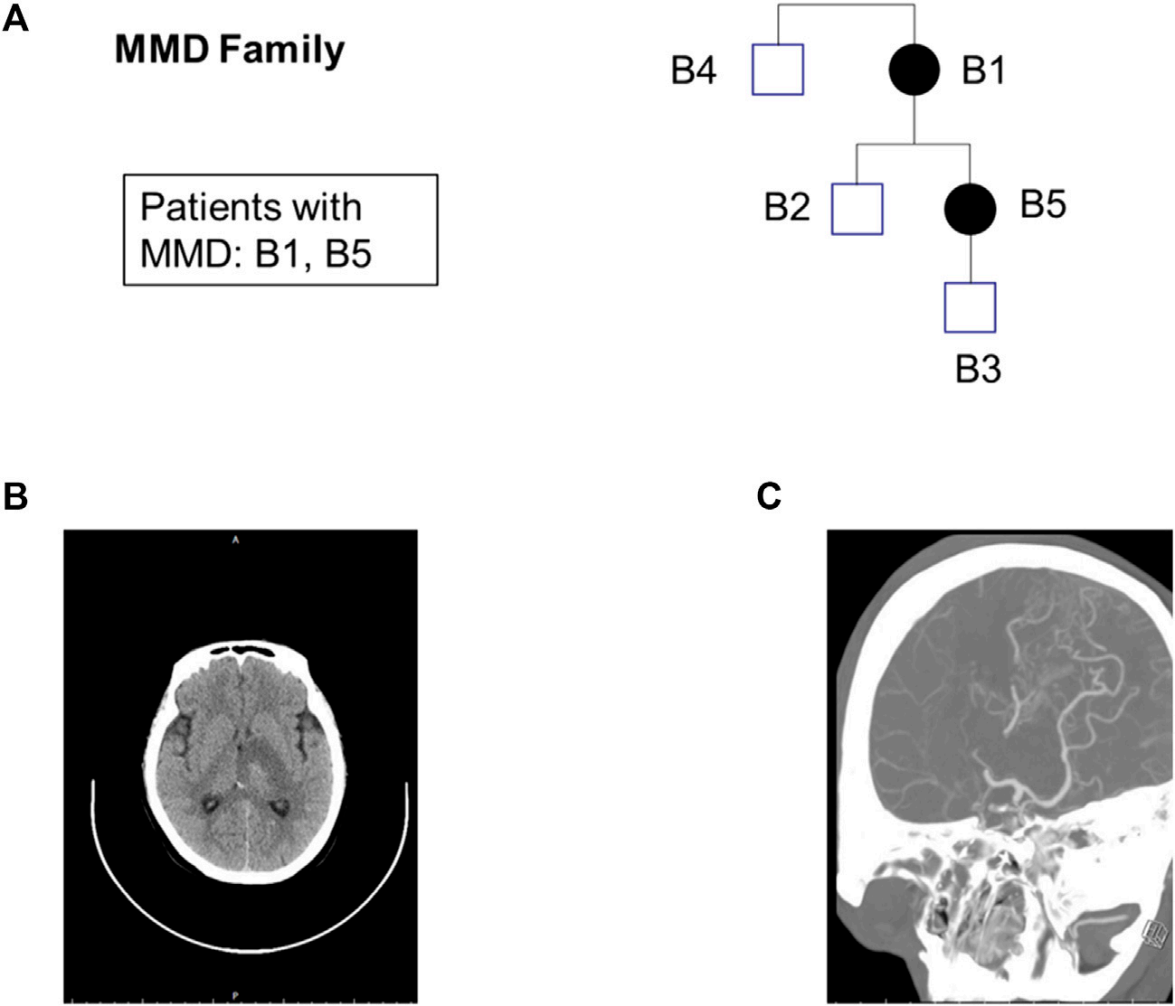
Exome sequencing maps for the MMD family. **(A)** Pedigree charts. Squares: male; circles: female; black-filled symbols: patients; **(B)** CT of patient B1; **(C)** CTA (Computed Tomography Angiography) of patient B1.

The pedigrees of five samples and the results of the patient’s CT and CAT tests are shown in Figure 1. The average sequencing depth of the five samples exceeded 100×, and the coverage of regions > 10× exceeded 99%. The number of SNVs and Indels obtained from each sample after data analysis are shown in Supplementary Table S4.

### Screening for Candidate Pathological Changes

**FIGURE 2:**
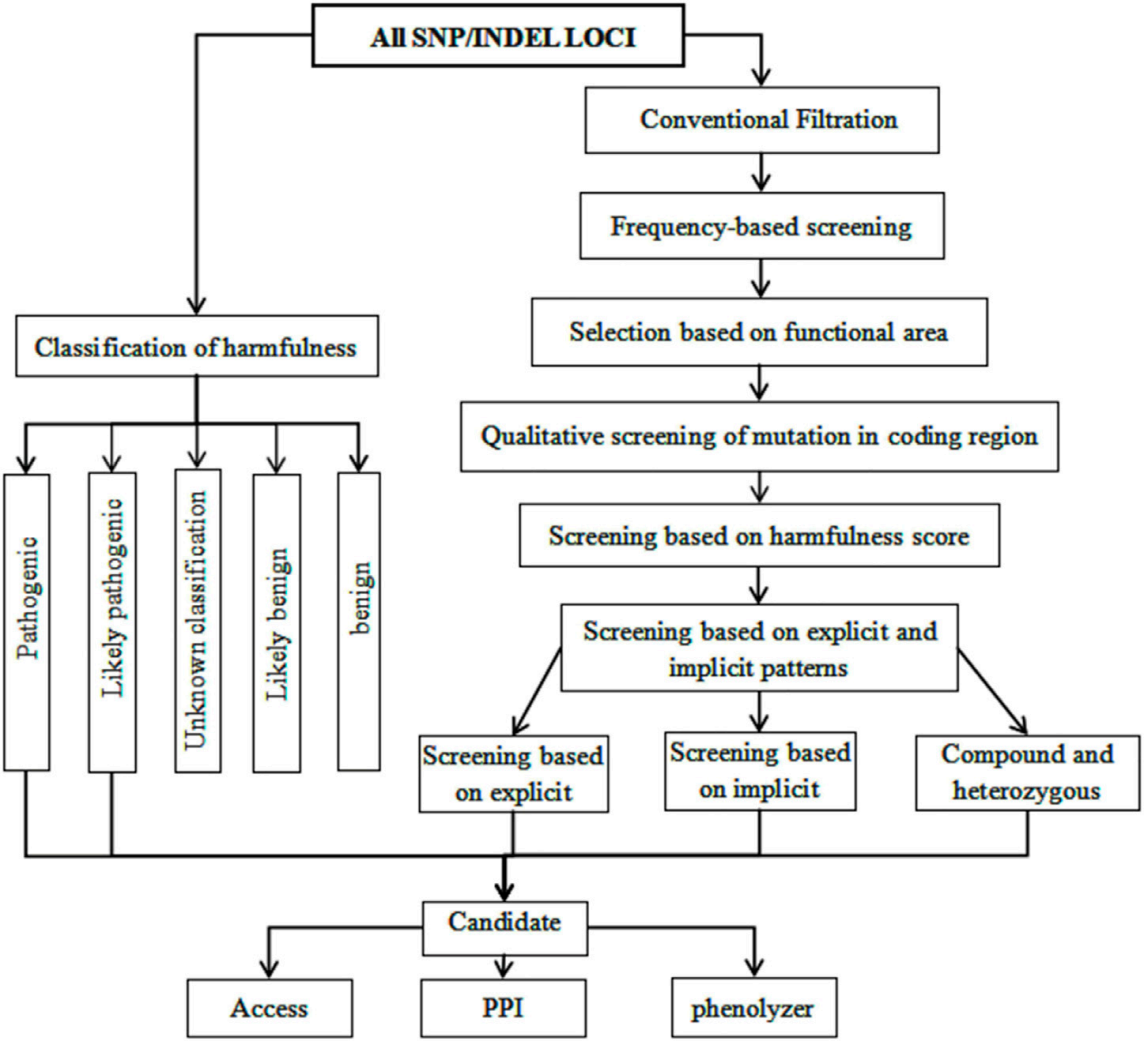
Analysis Flow Chart 2. Advanced analysis pipeline: Screening based on mutation sites and their harmfulness; Screening based on sample recessive patterns; Screening based on candidate genes and relationship with disease phenotypes; Pathway enrichment of candidate genes through GO and KEGG analysis (also using DisGeNet and Phenolyzer to analyze gene-disease phenotype associations).

Mutation loci were screened in accordance with the scores predicted by SIFT, PolyPhen, MutationTaster, and CADD. Candidate loci were further screened according to the process shown in Figure 2. The analysis identified multiple recessive pathogenic genes and notably, of these, loci mutation-related genes were within the *TTN* gene (rs771533925, rs559712998 and rs72677250) (Table 2).

**TABLE 2:** The detailed information of point mutation site.

Sample ID	Variant	RS ID	Gene	Coding DNA change	Protein change	Zygosity	ACMG	ExonicFunc	SIFT,Polyphen2_HVAR,Polyphen2_HDIV,MutationTaster,CADD
B1	2:179412799-C-T	rs771533925	TTN	c.G66359A; c.G88631A; c.G66935A;	p.R22245H; p.R29544H; p.R22312H; p.R22120H; p.R28617H	het	.	missense SNV	D/D/D/D/24.0
				c.G85850A; c.G66734A; c.G93554A					
B2	2:179412799-C-T	rs771533925	TTN	c.G66359A; c.G88631A; c.G66935A;	p.R22245H; p.R29544H; p.R22312H; p.R22120H; p.R28617H	het	.	missense SNV	D/D/D/D/24.0
				c.G85850A; c.G66734A; c.G93554A					
B1	2:179466289-C-T	rs559712998	TTN	c.G28615A; c.G47731A; c.G28816A;	p.V16838I; p.V9539I; p.V9606I; p.V9414I; p.V15911I	het	.	missense SNV	T/B/B/D/20.2
				c.G55435A; c.G50512A; c.G28240A					
B4	2:179466289-C-T	rs559712998	TTN	c.G28615A; c.G47731A; c.G28816A;	p.V16838I; p.V9539I; p.V9606I; p.V9414I; p.V15911I	het	.	missense SNV	T/B/B/D/20.2
				c.G55435A; c.G50512A; c.G28240A					
B5	2:179466289-C-T	rs559712998	TTN	c.G28615A; c.G47731A; c.G28816A;	p.V16838I; p.V9539I; p.V9606I; p.V9414I; p.V15911I	het	.	missense SNV	T/B/B/D/20.2
				c.G55435A; c.G50512A; c.G28240A					
B3	2:179476144-C-T	rs72677250	TTN	c.G24193A; c.G43108A; c.G23992A;	p.E15297K; p.E8065K; p.E7998K; p.E14370K; p.E7873K	het	.	missense SNV	T/P/D/D/23.7
				c.G45889A; c.G50812A; c.G23617A					
B5	2:179476144-C-T	rs72677250	TTN	c.G24193A; c.G43108A; c.G23992A;	p.E15297K; p.E8065K; p.E7998K; p.E14370K; p.E7873K	het	.	missense SNV	T/P/D/D/23.7
				c.G45889A; c.G50812A; c.G23617A					

### Validation of Candidate Loci by CRISPR-Cas12a

**FIGURE 3:**
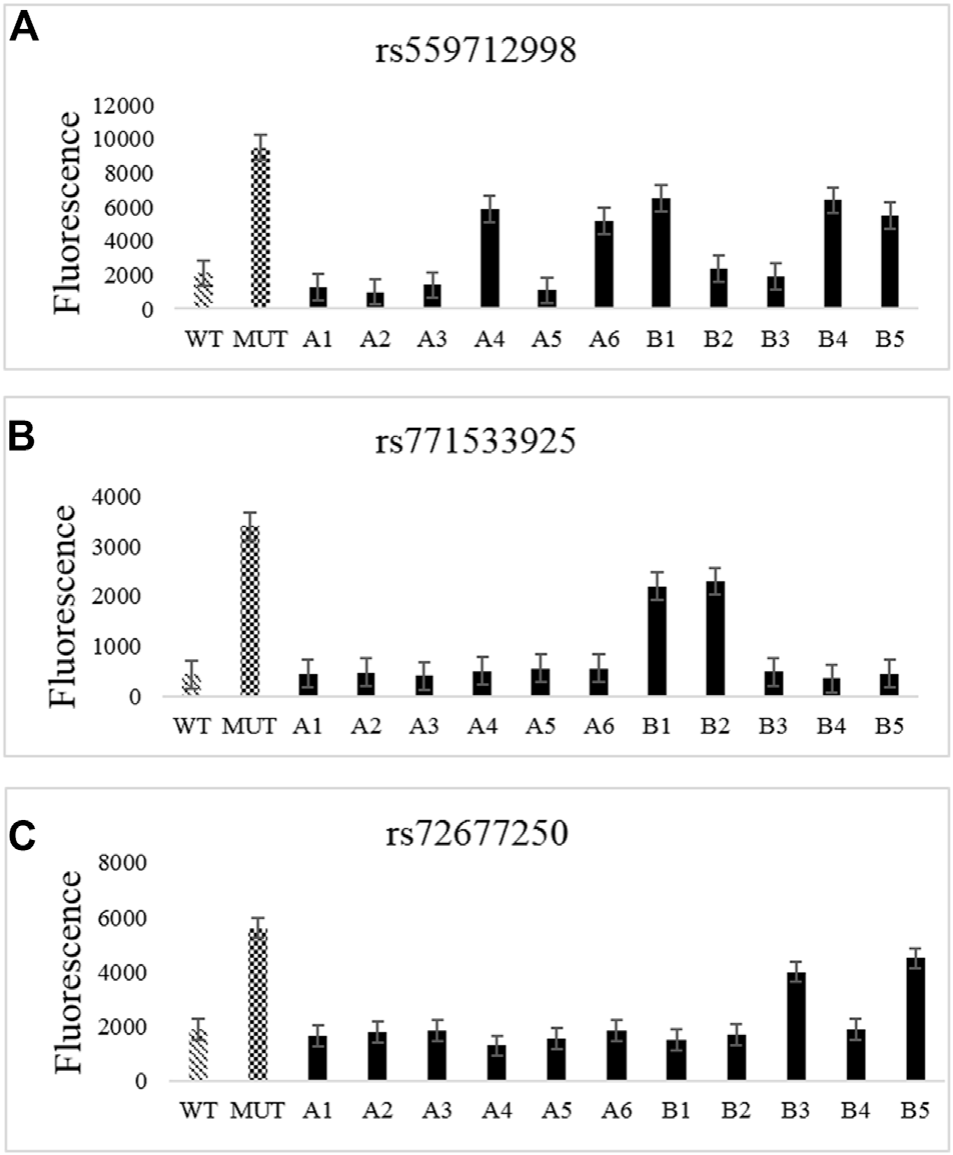
CRISPR-Cas12a analysis of *TTN* gene mutation loci in familial samples. **(A–C)** CRISPR-Cas12a test results for rs72677250 **(A)**, rs559712998 **(B)**, and rs771533925 **(C)**.

The test results obtained by the CRISPR-Cas12a system for mutation loci in the *TTN* gene in family samples (Figure 3) were consistent with those obtained from WES sequencing (Table 3), thus verifying the presence of mutations in the samples.

**TABLE 3:** Analysis of TTN Gene Mutation Results by CRISPR test and Sanger Sequencing in Family Samples.

TTN detection site		Sanger	CRISPR-Cas12a
		N = 5	N = 5
RS559712998	MUT	3 (60%)	3 (60%)
	WILD	2 (40%)	2 (40%)
RS771533925	MUT	2 (40%)	2 (40%)
	WILD	3 (60%)	3 (60%)
RS72677250	MUT	2 (40%)	2 (40%)
	WILD	3 (60%)	3 (60%)

### Validation of Candidate Loci by CRISPR-Cas12a in Sporadic Samples

**FIGURE 4:**
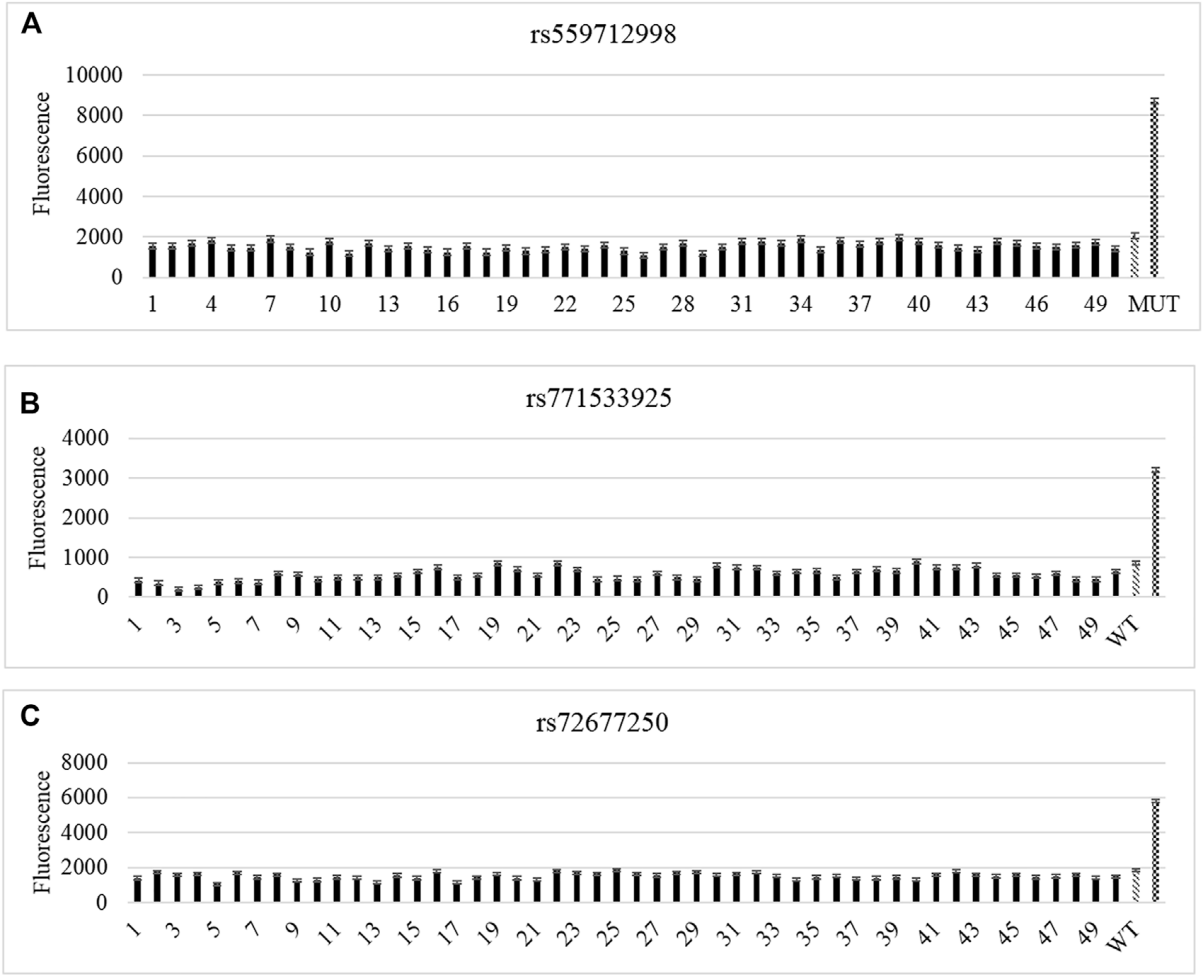
CRISPR-Cas12a analysis of *TTN* gene mutation loci in sporadic samples. **(A–C)** CRISPR-Cas12a test results for rs559712998, rs72677250 **(A),** rs771533925 **(B)**, and rs72677250 **(C)**.

Next, CRISPR-Cas12a system was used to test a total of 50 sporadic samples for gene mutations. No mutation was found at rs771533925, rs559712998 and rs72677250 of *TTN* gene in sporadic samples (Figure 4).

### The Deleterious Effects of rs771533925, rs559712998 and rs72677250

In addition, SIFT (Choi and Chan, 2015) PROVEAN (Vaser et al., 2016) and PolyPhen (Adzhubei et al., 2013) algorithms were used to predict the effects of amino acid substitutions on protein function (Table 4). All three databases showed that rs771533925 was potentially destructive. On the contrary, rs559712998 was considered tolerable according to these analyses. However, while rs72677250 was considered tolerable according to the SIFT database, it was considered to be potentially harmful according to the PROVEAN and PolyPhen databases.

**TABLE 4:** Hazard prediction of RS771533925, RS559712998 and RS72677250 mutations.

	Gene	PROVEAN prediction	SIFT prediction	Polyphen
rs771533925	TTN	Deleterious	Damaging	possibly_damaging
rs559712998	TTN	Neutral	Tolerated	benign
rs72677250	TTN	Deleterious	Tolerated	possibly_damaging

PROVEAN (Protein Variation Effect Analyzer) is a tool to predict whether biomolecular structure Variation affects Protein function; SIFT(sorts intolerant from tolerant) is a tool for predicting non-synonymous variations based on sequence homology; PolyPhen (Polymorphism Phenotyping) is a tool which predicts possible impact of an amino acid substitution on the structure and function of a human protein using straightforward physical and comparative considerations.

### *TTN* Mutation Sites rs72677250, rs559712998 and rs771533925 Global Population Frequency and Function Analysis

**FIGURE 5:**
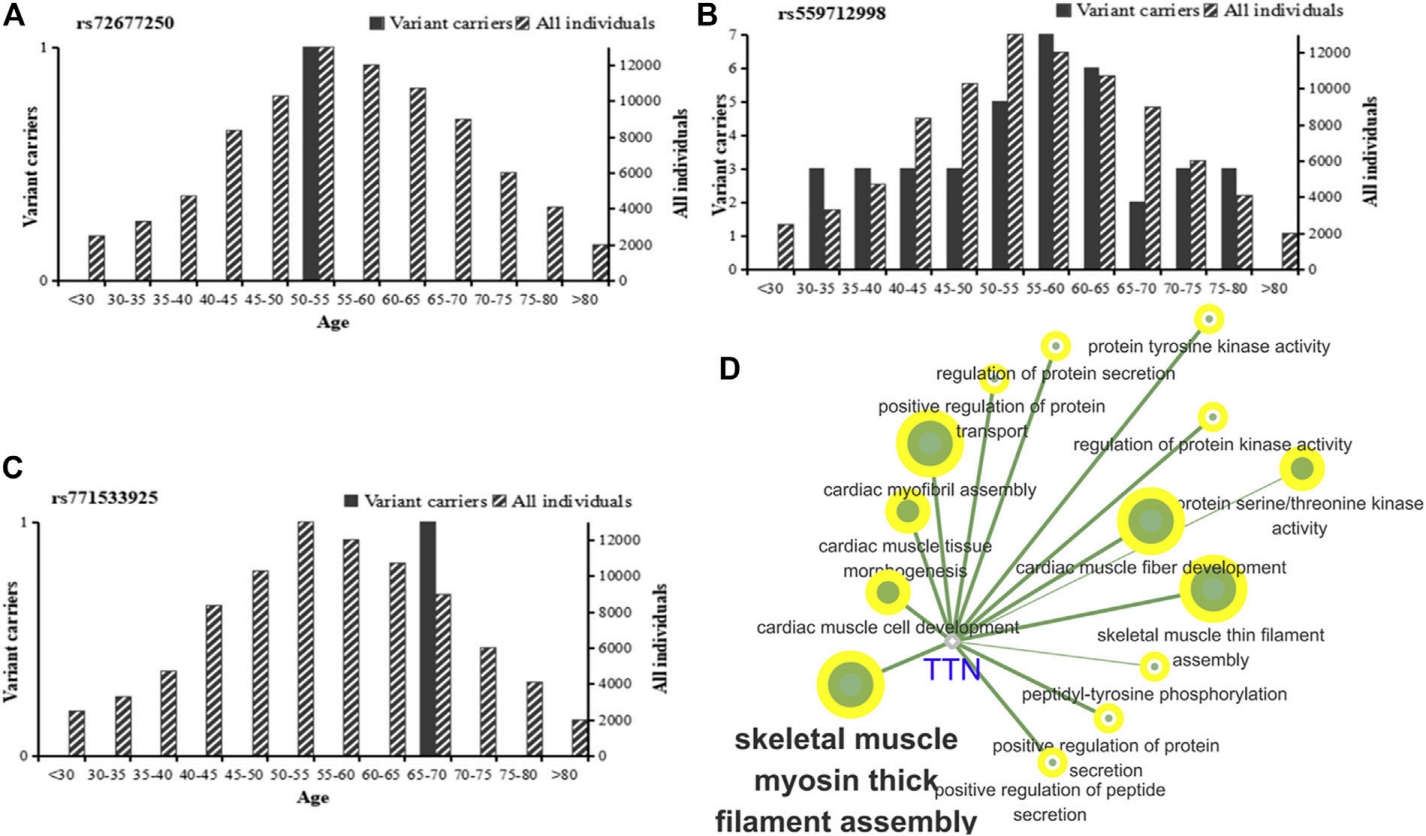
Global population frequency and function analysis of *TTN* mutation sites. **(A–C)** Global population frequencies among different age groups for the rs72677250 mutation **(A)**, rs559712998 mutation **(B)**, and rs771533925 mutation **(C)**. Analysis included heterozygous variant carriers, homozygous variant carriers. **(D)** GO functional enrichment analysis of *TTN* using a two-sided hypergeometric test with Bonferroni correction.

We analyzed the risk alleles (rs72677250, rs559712998 and rs771533925) in accordance with the EXAC database. We identified significant differences in frequency across the global population. The highest frequency of rs72677250 in the South Asian population was 0.00003269, the highest frequency of rs559712998 in the East Asian population was 0.002574, the highest frequency of rs771533925 in the East Asian population was 0.00005568, and the total frequency of rs559712998 mutations was 0.000192; the latter being the highest frequency of all three mutation sites (Table 5). According to age analysis of these three loci within the global population, we found that the rs72677250 mutation site was predominant in subjects aged 50–55 years, the rs559712998 mutation site was predominant in subjects aged 30–80 years, and the rs771533925 mutation site was predominant in subjects aged 65–70 years (Figures 5A–C). GO analysis was then conducted using Cytoscape 3.8.2 software with the ClueGO (Bindea et al., 2009) plugin, showing that the mutation locus for *TTN* were involved in a range of important biological processes, including myosin thick filament assembly in skeletal muscle, positive regulation of protein transport, serine/threonine kinase activity, and cardiac muscle fiber development (Figure 5D).

**TABLE 5:** Analysis of RNF213 and MMP3 gene mutation.

Gene	Genotype		Sanger sequencing results	Control group
			Liao cheng	
			Case group	
			(*n* = 34)	(*n* = 37)
RNF213	rs112735431	Mutation wild	8 (23.5%)	0 (0%)
			26 (76.4%)	37 (100%)
	*p* value		0.0019	
	rs148731719	Mutation	4 (11.8%)	3 (8.1%)
		wild	30 (88.2%)	34 (91.9%)
	*p* value		0.6082	
MMP3	6A6A		23 (67.6%)	2 (5.4%)
	5A6A		11 (32.4%)	35 (94.6%)
	5A5A		0 (0%)	0 (0%)
	*p* value		0.00001	
	6A allele frequency		57 (83.8%)	39 (52.7%)
	5A allele frequency		11 (16.2%)	35 (47.3%)
	*p* value		0.0001	

## Discussion

Moyamoya disease is a chronic and progressive disease that can cause cerebral ischemia, cerebral infarction, cerebral hemorrhage, etc., which is a great harm to patients (Kuroda and Houkin, 2008). Patients suffering from moyamoya disease generally could not heal themselves without scientific treatment, and even the condition may continue to aggravate, causing irreversible harm, and bringing great economic burdens to patients and their families to a certain extent (Zhang et al., 2022).

Screening family genetic patients to obtain new or known gene mutations, whole-exome sequencing has the advantages of accuracy and comprehension (Zhang et al., 2021). However, whole-exome sequencing has drawbacks such as time-consuming and high cost, which is not conducive to the large-scale screening of samples. On this basis, the CRISPR technology is used to detect new or known disease-causing gene loci, filling the blank of large-scale sample screening in terms of gene sequencing.

The CRISPR-Cas system can recognize foreign DNA or RNA, directing cleavage to silence the expression of the foreign gene (Brouns et al., 2008; Marraffini and Sontheimer, 2008; Garneau et al., 2010). It can be identified as an efficient gene editing tool for its precise targeting ability (Nelles et al., 2016). Studies have indicated that a diagnostic platform based on CRISPR-Cas represents an exciting prospect for the detection of cancer and genetic diseases (Mali et al., 2013). Cas12a (cpf1) is a new type of programmable DNA enzyme found in the CRISPR system (Zhou et al., 2014). In the presence of specific directing crRNA, Cas12a also has the ability to cut non-target DNA after cleavage of the target DNA (Gilbert et al., 2013; Qi et al., 2013). Therefore, the CRISPR-Cas12a system can be more effective for *in vitro* detection by adding a fluorescent DNA reporter (Mohanraju et al., 2016; Nelles et al., 2016; Koonin et al., 2017) which can emit detectable fluorescence after cleavage. This provides a fluorescence-based assay which only requires low technology instrumentation such as a microplate reader to provide quantitative measurements of mutations.

The rs112735431 and rs148731719 mutations in the *RNF213* gene are known to be associated with the pathogenesis of MMD in Chinese subjects (Liu et al., 2011; Morito et al., 2014; Kobayashi et al., 2015; Hu et al., 2017). *RNF213* is located on human chromosome 17 (the 17q25.3 region) and its expression occurs in different organs (Kuriyama et al., 2008). An imbalance leads to vascular smooth muscle hyperplasia and thickening, thus leading to vascular stenosis, one of the key pathogenic factors responsible for MMD (Li et al., 2010). Additionally, other studies have shown that the 1171 (6A/6A) mutation in the *MMP3* gene is associated with heightened MMD susceptibility with the risk of the (6A/6A) genotype being higher than the (5A/6A) genotype (Wang et al., 2013; Ma and You, 2015; Wang X. et al., 2020). Preliminary experiments analyzed rs112735431 and rs148731719 mutations in the *RNF213* gene in MMD patients and healthy control subjects. In the pre-experiment, we discovered that it was the rs112735431 *RNF213* gene mutation but not the rs148731719 mutation affecting the occurrence and development of MMD. At present, Sanger sequencing is mostly carried out for cerebrovascular diseases, and CRISPR technology is rarely studied. Therefore, we first used CRISPR-Cas12a system to compare the technical feasibility. The results showed that rs112735431 and rs148731719 mutations of the RNF213 gene were successfully detected by the CRISPR-Cas12a system with 100% agreement with the results of Sanger sequencing.

In this study, we performed WES on five family members of the MMD family to identify MMD genetic-related mutation loci, establishing a new candidate susceptibility loci in the *TTN* gene. We also detected mutant loci in MMD patients and healthy controls to investigate differences in the mutation loci across the population using CRISPR-Cas12a assays. Then, we compared CRISPR-Cas12a technology with Sanger sequencing and WES for the detection of mutations to highlight the diagnostic efficacy of CRISPR-Cas12a. Finally, we conducted the analysis of population frequency, harmfulness, and functional enrichment on *TTN*.

Our WES analysis also identified a number of recessive pathogenic genes in five members of two MMD families. The *TTN* was identified as the gene containing mutation-related loci (rs771533925, rs559712998 and rs72677250). The EXAC database was used to analyze the risk alleles (rs72677250, rs559712998 and rs771533925). Significant differences were identified in the frequencies of these alleles across the global population. Based on PROVEAN, SIFT, and PolyPhen algorithms, rs771533925 and rs72677250 were considered to be potentially damaging in all three databases where rs559712998 was considered to be tolerable in contrast. GO analysis showed that the targets of *TTN* were involved in many important biological processes. Together with actin and myosin, *TTN* constitute an important component of human cardiac muscle and skeletal muscle. Interestingly, serum antibodies directed against *TTN* were found in patients with melanoma-associated retinopathy, suggesting *TTN* was a potential biomarker for melanoma and also an association with carcinogenesis. Future studies should address the role of *TTN* gene mutations in the pathogenesis of MMD.

In the present study, CRISPR–Cas12a was developed as a novel assay that could sensitively and specifically detect MMD mutation gene loci. Moreover, compared with Sanger sequencing, the CRISPR-Cas12a method is easier, cheaper, and more sensitive for single gene mutations, so it should be promoted to use widely. Also, CRISPR-Cas12a assays were conducted to detect mutations in the candidate genes within the MMD family. Similarly, SNP loci within the *TTN* gene were readily detected with results consistent with the WES analysis. Further detection of mutations in the *TTN* gene in 50 clinical samples revealed that there was no mutation in the *TTN* gene SNP loci and no recessive genetic risk for loci mutations. We speculated that mutations at the *TTN* locus may play an important role in the familial inheritance of MMD. However, our data is limited and a large number of samples are still needed to verify. What’s more, these mutations are likely suitable for identifying patient pedigrees and assessing the genetic risk of MMD in large-scale screening.

## Conclusion

Our study identified *TTN*, a new specific candidate gene in familial moyamoya disease. We also established that CRISPR-Cas12a assays, which can effectively detect MMD mutations, and with significant advantages in time, suggest utility in the rapid detection of MMD mutations. Furthermore, with the detection technology embedded within the reagents, the instrumentation required is comparatively easy, proposing the CRISPR-Cas12a system could be readily developed as accurate, portable diagnostic tests for MMD. Therefore, the CRISPR-Cas12a system can be used to overcome obstacles created by previous platforms and provide a highly sensitive and convenient detection system for MMD mutations with DNA acquired from clinical blood samples.

## Data Availability

The datasets presented in this study can be found in online repositories. The names of the repository/repositories and accession number(s) can be found in the article/Supplementary Material.

## Supplementary Material

Supplementary Figure S1 Analysis flow chart 1. Overview of the information analysis process in three parts: Sequencing data quality assessment to determine whether library sequencing meets the required standards; Variation detection in the sample, with statistics and annotations performed for the detected variations; Variation screening and prediction of disease relevance based on the results of variation detection to identify harmful mutation sites or genes related to disease.

Supplementary Figure S2 CRISPR-Cas12a analysis of *TTN* gene mutation loci. **(A-D)** CRISPR-Cas12a test results for rs559712998 and rs771533925 mutation type **(A,B)**; rs559712998 and rs771533925 wild type **(C, D)**.

Supplementary Figure S3 Validation of the CRISPR-Cas12a detection system. **(A and B)** Validation of the CRISPR-Cas12a detection system.

Supplementary Figure S4 Sequencing peak map of RNF213 and MMP3. **(A, B)** Sequencing peak maps of the RNF213 **(A)** and MMP3 **(B)** genes.

## Abbreviations

MMD, Moyamoya disease; RNF213, ring finger protein 213; PAM, protospacer adjacent motif; PCR, polymerase chain reactions; IRB, Institutional Review Board; IBD, identity-by-descent; SNVs, Single nucleotide variants; gVCF, Genome VCF; CNVs, Copy number variants; ACMG, American College of Medical Genetics and Genomics; CT, computed tomography; CTA, Computed Tomography Angiography; WES, whole-exome sequencing.
