# Supplementary figures and images for: CRISPR Detection and Research on Screening Mutant Gene TTN of Moyamoya Disease Family Based on Whole Exome Sequencing
Source: Front Mol Biosci. 2022 Mar 9;9:846579. doi: 10.3389/fmolb.2022.846579 (PMC8959584; doi:10.3389/fmolb.2022.846579)

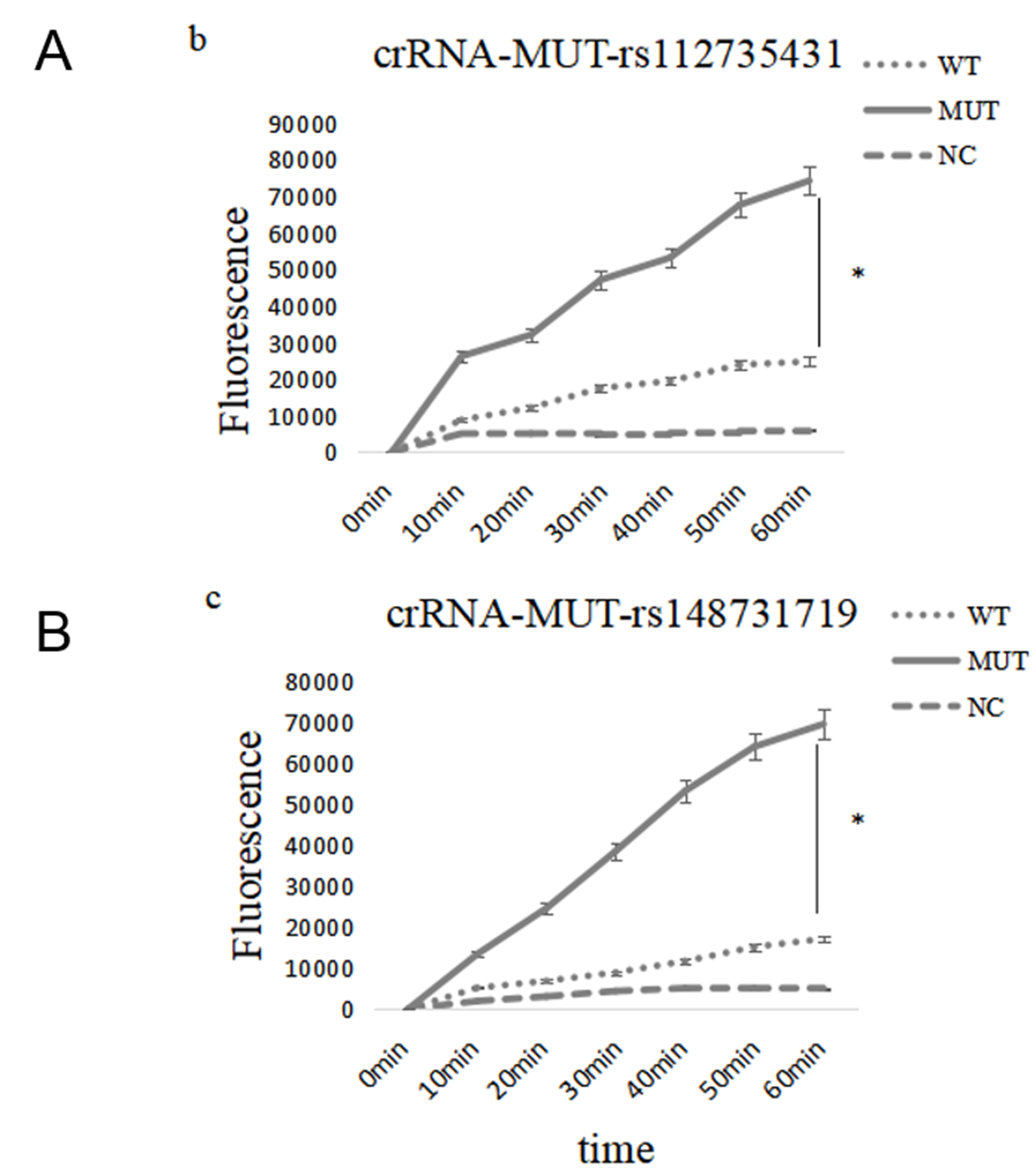

Supplement: Supplementary file 1 [file Image4.TIF]

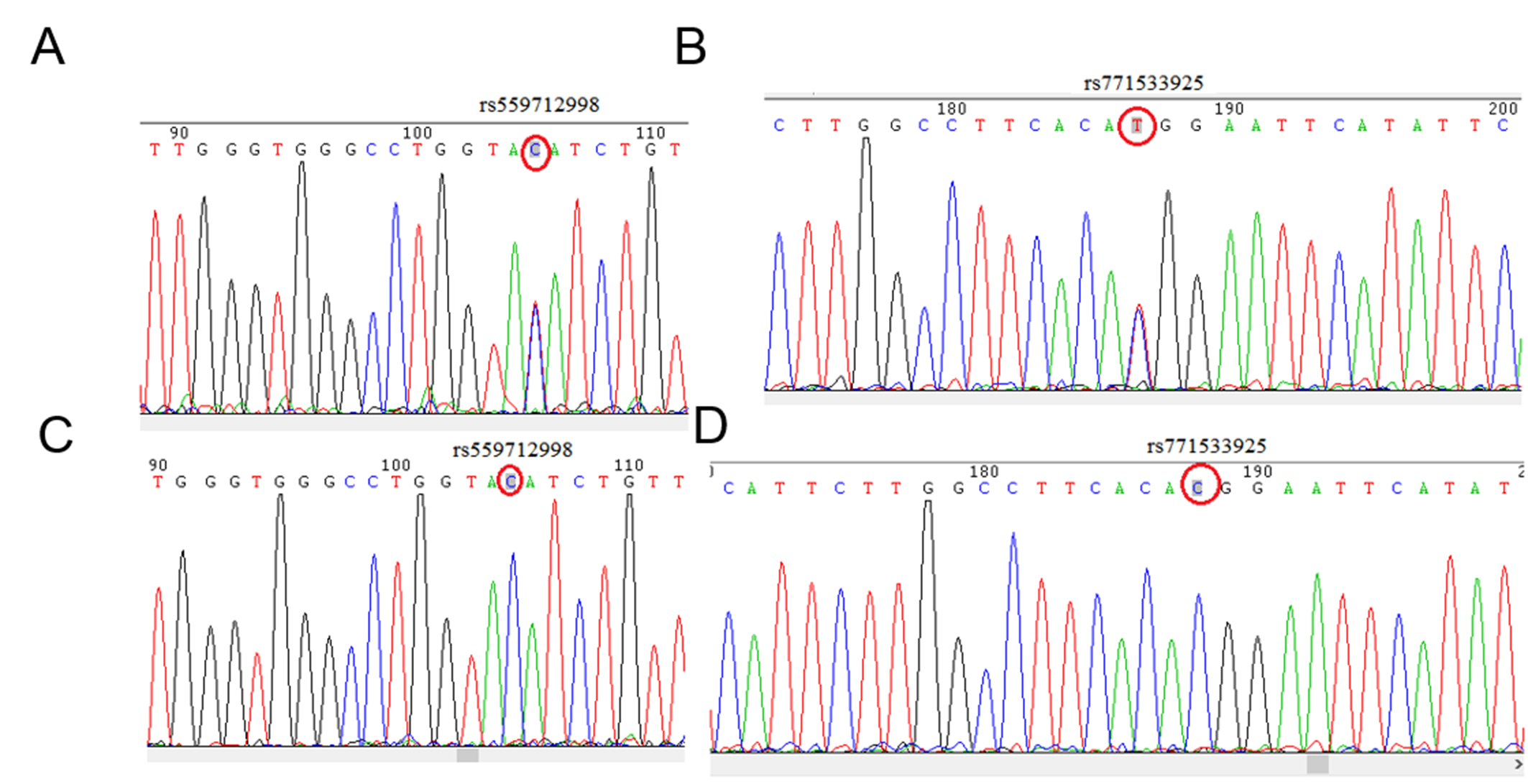

Supplement: Supplementary file 2 [file Image2.TIF]

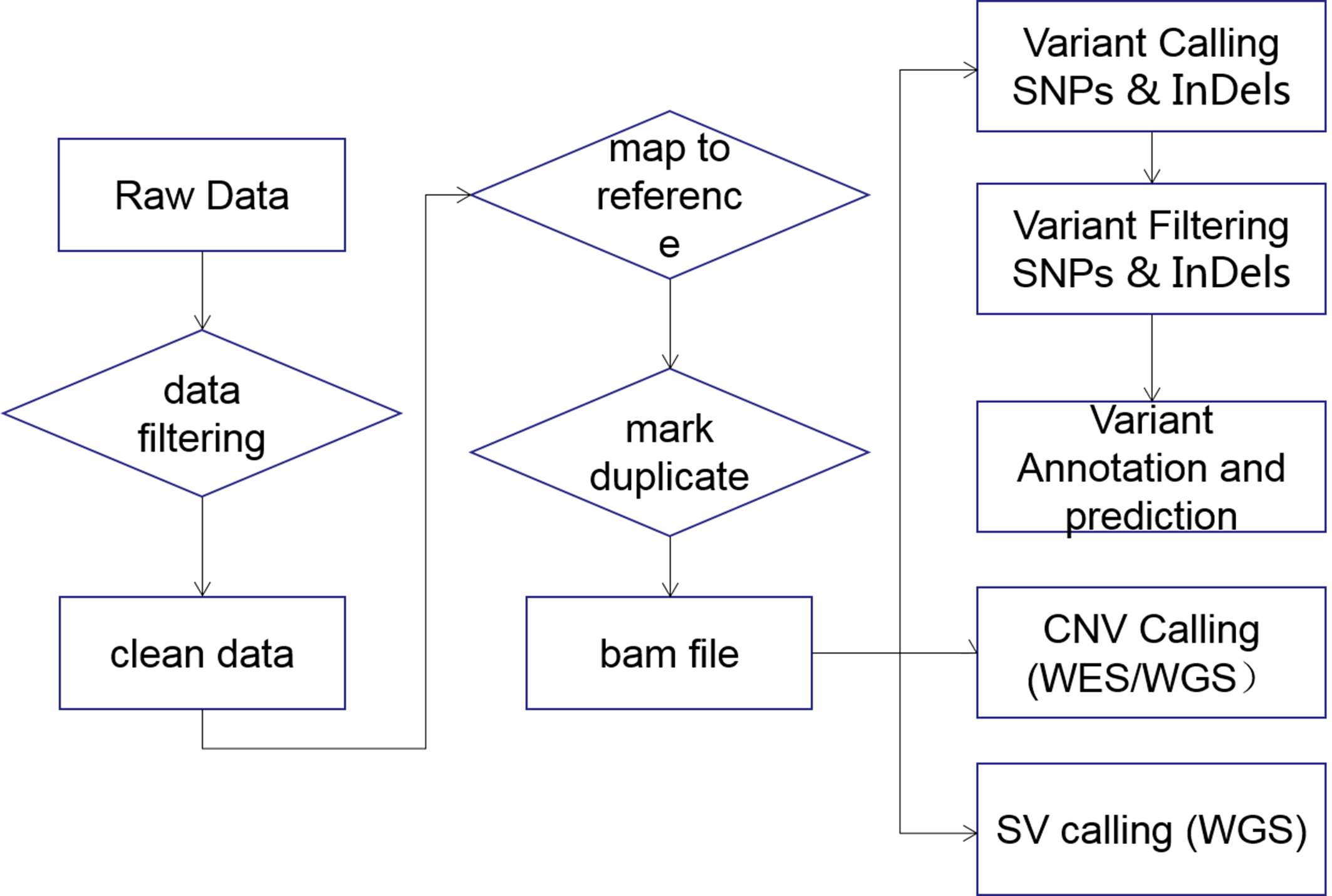

Supplement: Supplementary file 3 [file Image1.TIF]

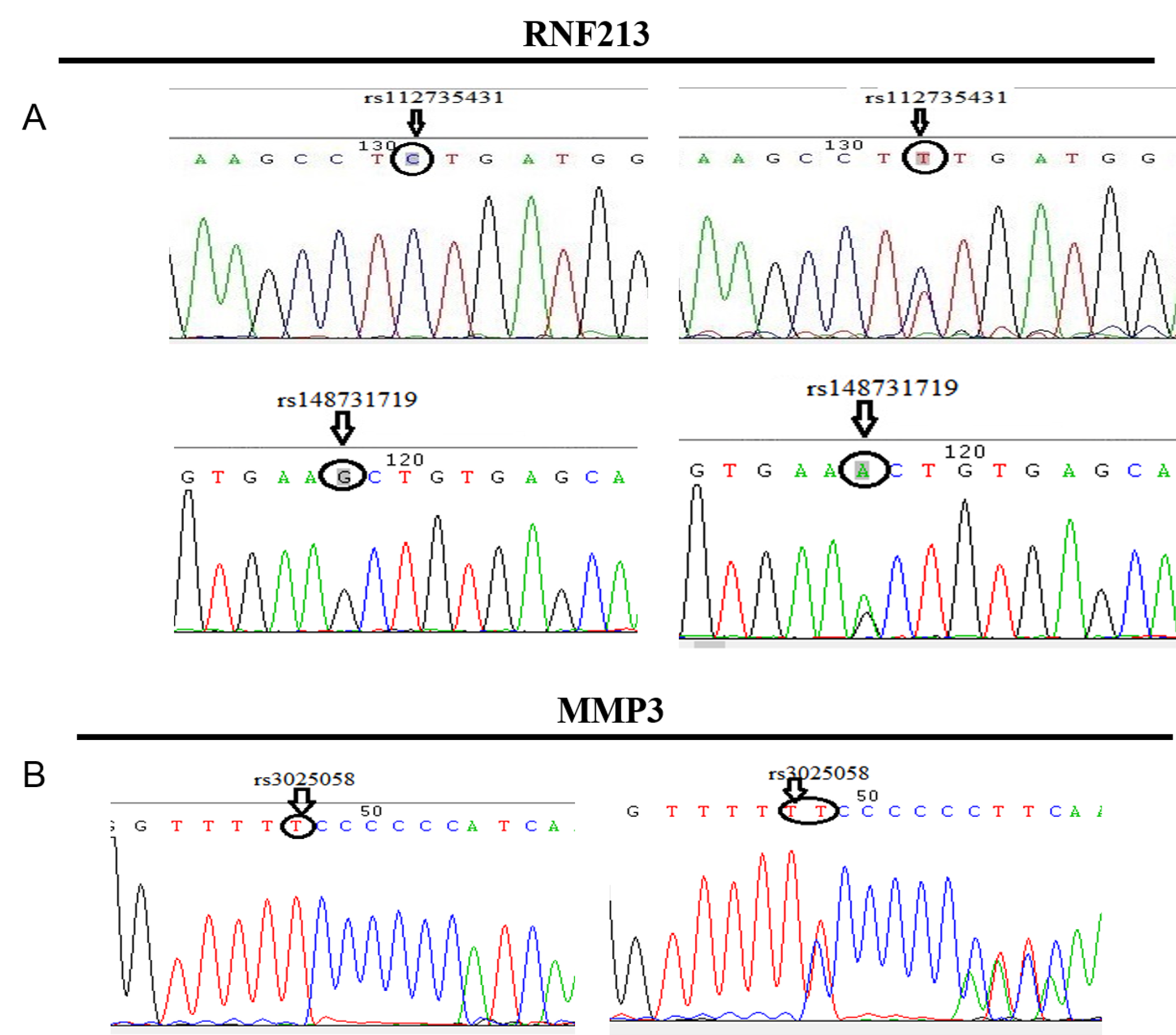

Supplement: Supplementary file 4 [file Image5.TIF]
