# Supplementary material for: CRISPR Detection and Research on Screening Mutant Gene TTN of Moyamoya Disease Family Based on Whole Exome Sequencing
Source: Front Mol Biosci. 2022 Mar 9;9:846579. doi: 10.3389/fmolb.2022.846579 (PMC8959584; doi:10.3389/fmolb.2022.846579)
Supplement: Supplementary file 5 [file DataSheet1.docx]

**Table S1 Primers to be Synthesized**

| **Desired primers** | **Sequence (Primers were synthesized by Tianyi Huiyuan Biotechnology Co., Ltd.)** |
| --- | --- |
| cr-T7-F | 5`-GAAATTAATACGACTCACTATAGGG-3` |
| CrDNA-R4810K-R | 5`-CAGCTCCATCCAAGGCTTCCTCAGATCTACAAGAGTAGAAATTACCCTATAGTGAGTCGTATTAATTTC-3` |
| CrDNA-A4399T-R | 5`-TGTCAGCAATGTGACACTGTGAGATCTACAAGAGTAGAAATTACCCTATAGTGAGTCGTATTAATTTC-3` |
| CrDNA-rs771533925-R | GCCTGGTATATCTCTTGATCTACAAGAGTAGAAATTACCCTATAGTGAGTCGTATTAATTTC-3` |
| CrDNA-rs559712998-R | ATCATTCTTGGCCTTCAGATGATCTACAAGAGTAGAAATTACCCTATAGTGAGTCGTATTAATTTC -3` |
| CrDNA-rs72677250 -R | AGTCTCTAAAGTCTACCGCACATCTACAAGAGTAGAAATTACCCTATAGTGAGTCGTATTAATTTC-3` |
| R4810K-F | 5`-GTCTCGCAGCCAGTCTCAAAGTCTGTAACCC-3` |
| R4810K-R | 5`-CCTCCCCTCCGCCCCACCCTGTTCCCCTAT-3` |
| A4399T-F | 5`-GTGTATTTGGTGTCAGTGTATGTCTAAGAGCA-3` |
| A4399T-R | 5`-CTGTCACCGTTCCATCAAGGTTGCTGTCACT-3` |
| MMP3-F | 5`-GAGGTTTCACTATGTTGCCCAGGCTGGTTTCG-3` |
| MMP3-R | 5`-TCACTGCCACCACTCTGTTCTCCTTGTCCTCA-3` |
| rs771533925-F | 5`-CTTTAAGTCTCATTTCTTCCAGTTTCCATGTTA-3` |
| rs771533925-R | 5`-GAGCGTCTTGTTGAGAAAACTGAATATGAAT-3` |
| rs559712998-F | 5`-CTTTTCCATCAATAGTCCTCTTCTCAATAACATAG-3` |
| rs559712998-R | 5`-CTTTGAAAGTTGCTACAGAATAAAAACAAATTAT-3` |
| rs72677250 -F | 5`-TTGCCTGTAAGATATCATTCAAAAGAGCAAAAA-3` |
| rs72677250 -R | 5`-ATAGGATACCATGTTGAAATGTGTCCAGTAG-3` |
| Probe | 5`-HEX-CTCACTACAGACGCACGCTA-BHQ1-3` |

**Table S2 Statistics of SNVs and Indels in all Samples**

| Sample | Mutation type | all | genotype.Het | genotype.Hom | novel | novel_proportion |
| --- | --- | --- | --- | --- | --- | --- |
| A1 | SNV | 362636 | 198455 | 164181 | 2315 | 0.006383812 |
|  | InDel | 59146 | 34505 | 24641 | 7697 | 0.130135597 |
| A2 | SNV | 343942 | 188870 | 155072 | 2163 | 0.006288851 |
|  | InDel | 54081 | 31442 | 22639 | 6994 | 0.129324532 |
| A3 | SNV | 304507 | 167565 | 136942 | 1944 | 0.00638409 |
|  | InDel | 49270 | 28382 | 20888 | 6250 | 0.12685204 |
| A4 | SNV | 349396 | 190659 | 158737 | 2206 | 0.006313753 |
|  | InDel | 53621 | 29473 | 24148 | 5968 | 0.111299677 |
| A5 | SNV | 313005 | 172353 | 140652 | 1969 | 0.006290634 |
|  | InDel | 49311 | 28690 | 20621 | 6167 | 0.125063373 |
| A6 | SNV | 464903 | 252872 | 212031 | 2929 | 0.006300239 |
|  | InDel | 73202 | 42469 | 30733 | 9157 | 0.125092211 |
| A7 | SNV | 354732 | 194862 | 159870 | 2219 | 0.006255427 |
|  | InDel | 57061 | 33175 | 23886 | 7217 | 0.126478681 |
| B1 | SNV | 388336 | 212851 | 175485 | 2409 | 0.006203391 |
|  | InDel | 60015 | 34744 | 25271 | 7634 | 0.127201533 |
| B2 | SNV | 456327 | 245811 | 210516 | 2860 | 0.006267435 |
|  | InDel | 70277 | 39866 | 30411 | 8855 | 0.126001394 |
| B3 | SNV | 296698 | 162969 | 133729 | 1846 | 0.006221815 |
|  | InDel | 43918 | 24028 | 19890 | 4639 | 0.105628672 |
| B4 | SNV | 305626 | 165855 | 139771 | 1950 | 0.006380347 |
|  | InDel | 47040 | 26930 | 20110 | 5909 | 0.125616497 |
| B5 | SNV | 453834 | 247701 | 206133 | 2809 | 0.006189488 |
|  | InDel | 70443 | 40609 | 29834 | 8304 | 0.117882543 |

Note:

(1) Sample: Sample name

(2) all: Number of all SNV/InDels

(3) genotype.Het: SNV/InDel number of heterozygous genotypes

(4) genotype.Hom: SNV/InDel number of homozygous genotypes

(5) novel: New SNV/InDel not annotated by dbSNP

(6) novel_proportion: Proportion of new SNV/InDel
